# Lower Thalamic Blood Flow Is Associated With Slower Stride Velocity in Older Adults

**DOI:** 10.3389/fnagi.2020.571074

**Published:** 2020-09-18

**Authors:** Noah D. Koblinsky, Sarah Atwi, Ellen Cohen, Nicole D. Anderson, Carol E. Greenwood, Bradley J. MacIntosh, Andrew D. Robertson

**Affiliations:** ^1^Rotman Research Institute, Baycrest Health Sciences, University of Toronto, Toronto, ON, Canada; ^2^Hurvitz Brain Sciences, Sunnybrook Research Institute, University of Toronto, Toronto, ON, Canada; ^3^Heart and Stroke Foundation Canadian Partnership for Stroke Recovery, Sunnybrook Research Institute, University of Toronto, Toronto, ON, Canada; ^4^Department of Medical Biophysics, University of Toronto, Toronto, ON, Canada; ^5^Department of Physical Therapy, University of Toronto, Toronto, ON, Canada; ^6^Departments of Psychology and Psychiatry, University of Toronto, Toronto, ON, Canada; ^7^Department of Nutritional Sciences, University of Toronto, Toronto, ON, Canada

**Keywords:** brain aging, arterial spin labeling, gait, pallidum, putamen, thalamus

## Abstract

**Background:**

Gait deficits are associated with brain atrophy and white matter hyperintensities (WMH) – both markers of underlying cerebral small vessel disease (SVD). Given reduced subcortical cerebral blood flow (CBF) is prevalent in SVD, we tested the hypothesis that regional CBF is positively associated with gait performance among older adults.

**Methods:**

Thirty-two older adults (55–80 years) with at least one vascular risk factor were recruited. We assessed gait during 2 consecutive walking sequences using a GAITRite system: (1) at a self-selected pace, and (2) while performing a serial subtraction dual-task challenge. We quantified CBF using pseudo-continuous arterial spin labeling MRI within 4 regions of interest: putamen, pallidum, thalamus, and hippocampus. We investigated associations between gait characteristics and overall CBF adjusting for age, sex, and height in an omnibus approach using multivariate analysis of variance, followed by regression analysis with each individual region. We also conducted further regression analyses to investigate associations between gait characteristics and frontal lobe CBF. Sensitivity analyses examined how the observed associations were modified by WMH, executive function, and depressive symptoms. A change of 10% in the model’s adjusted r^2^ and effect size was considered as a threshold for confounding.

**Results:**

Overall subcortical CBF was not associated with self-paced gait. When examining individual ROI, gait velocity was directly related to thalamic CBF (*p* = 0.026), and across all gait variables the largest effect sizes were observed in relation to thalamic CBF. In the dual-task condition, gait variables were not related to CBF in either the omnibus approach or individual multiple regressions. Furthermore, no significant associations were observed between frontal CBF and gait variables in either the self-paced or dual-task condition. Sensitivity analyses which were restricted to examine the association of velocity and thalamic CBF identified a cofounding effect of depressive symptoms which increased the effect size of the CBF-gait association by 12%.

**Conclusion:**

Subcortical hypoperfusion, particularly in regions that comprise central input/output tracts to the cortical tissue, may underlie the association between gait deficits and brain aging.

## Introduction

Slowing of gait, cognitive decline, and mood disorders are aging-related conditions that predict frailty and functional dependence. Cerebral small vessel disease (SVD) may be a driver of these behavioral changes by disrupting the interaction between frontal-subcortical circuits, the basal ganglia, and the thalamus which regulates motor and cognitive functions (Schmahmann and Pandya, 2008). The SVD disconnection syndrome theory posits that subcortical vascular lesions, a hallmark of SVD, can exert a direct influence on frontal-subcortical mediated brain functions (Ter Telgte et al., 2018). Broadly speaking, aging and cardiovascular risk factors lead to vascular remodeling, which alters cerebral hemodynamics and can impact brain metabolism or the autoregulatory processes that protect the brain (Pugh and Lipsitz, 2002; Pantoni, 2010). Cerebrovascular changes are a common finding, occurring in up to one-half of older adults (Gorelick et al., 2011); understanding their effects is an important step toward developing strategies to slow and prevent functional decline.

Slower gait velocity and increased stride variability are indicators of fall risk, which can lead to fractures, hospitalization, and death. There is a 30% risk of a fall per annum among adults over 65 years of age (Bergen et al., 2016). Measuring gait performance is an informative and efficient behavioral neurology assessment (Rosano et al., 2008; Studenski et al., 2011) and can serve as a probe of cognitive function, especially when challenged with a secondary (dual) task. Differences between self-paced and dual-task gait (also known as dual-task cost) are said to highlight the role of executive function (van Iersel et al., 2008) and frontal brain region activity (Yogev-Seligmann et al., 2008) in gait. Dual-task cost is more pronounced in those with cognitive deficits, and has shown to be an independent predictor of falls, even in those with relatively healthy gait.

An important characteristic of SVD is impaired cerebral hemodynamics. In addition to a greater white matter hyperintensity (WMH) burden, lower cerebral blood flow (CBF) in older adults is associated with reductions in brain tissue volume and impaired cognitive function and attention (Bertsch et al., 2009; Zonneveld et al., 2015). Subcortical networks linked to executive function and gait lie at the perfusion boundaries of vascular territories and may be more susceptible to fluctuations in cerebral perfusion (Pugh and Lipsitz, 2002; Pantoni, 2010). The pallidum, putamen, and thalamus are subcortical brain regions involved in motor movement networks and are typically perfused by arterial branches of the middle cerebral artery (MCA) which run deep into the subcortical tissues (Roland, 1984). These structures may affect gait and posture control through involvement in coordinated automatic gait processes linked to the brain stem, as well as more complex motor and cognitive processes associated with the frontal cortex (Takakusaki, 2017), specifically dual-task gait. Cerebrovascular changes observed in these subcortical regions have been linked to gait disturbances in the elderly (de Laat et al., 2010). In addition, the hippocampus, although not considered a truly subcortical structure or motor control region, has shown to be involved in frontal-subcortical functions in relation to spatial navigation and gait (Lithfous et al., 2013; Beauchet et al., 2015).

Gait speed and lower extremity function are directly associated with executive task-related changes in both cerebral blood velocity (Doppler ultrasound) and blood oxygenation level dependent cerebral hemodynamics (magnetic resonance imaging; MRI) within the MCA perfusion territory (Jor’dan et al., 2017). Furthermore, lacunar infarcts and WMH – telltale signs of chronic cerebrovascular impairment – are independently associated with gait speed, gait variability, and falls (Srikanth et al., 2009; de Laat et al., 2010). Yet, no studies to date have examined relationships between features of gait and CBF in corresponding brain regions involved in motor execution. The aim of this study was to investigate cross-sectional associations between resting CBF in subcortical regions involved in gait and the spatial and temporal characteristics of gait. In addition, the relationship between frontal CBF and gait function was assessed, which may be particularly sensitive to dual-task performance. We hypothesized that regional CBF would be positively correlated with gait speed and inversely correlated with gait variability under self-paced and dual-task conditions. Sensitivity analyses were conducted to explore how these associations were modified by WMH, executive function, and depressive symptoms.

## Materials and Methods

### Participants

We recruited older adults (55–80 years) with one or more vascular risk factors (e.g., hypertension, type II diabetes mellitus, dyslipidemia) and a sedentary lifestyle (<90 min of moderate or vigorous intensity physical activity per week) through community and family medicine advertising. Exclusion criteria included an inability to walk independently for 10 meters, a history of carotid artery stenosis (>70%), gray matter stroke, cardiopulmonary disease, diabetes requiring insulin therapy, dementia diagnosis, Montreal Cognitive Assessment (MoCA) score below 21, and contraindications to MRI. The local research ethics board approved the study (227–2014) and the work was carried out in accordance with The Code of Ethics of the World Medical Association (Declaration of Helsinki) for experiments involving humans. All participants provided informed written consent.

### Gait Assessment

We quantified gait characteristics using the GAITRite^®^ Classic system – a 5.73 m long pressure sensitive walkway that measures spatial and temporal parameters of gait. GAITRite^®^ is a reliable and valid tool for calculating gait characteristics in healthy and cognitively impaired older adults, and under dual-task conditions (Bilney et al., 2003). Participants walked without a gait aid along the walkway at: (1) their self-selected “normal” pace, and (2) under a dual-task condition of serial subtractions (i.e., spoken answers starting at 100 and subtracting by 7). Three trials of each condition were recorded and averaged for analysis. Each walking trial began and ended approximately one meter from the walkway to minimize acceleration and deceleration influences. We calculated four gait metrics: stride velocity (cm/s), stride length (cm), stride time (s), and stride time variability (%). Stride time variability was expressed as the coefficient of variation and calculated by dividing the standard deviation in stride time across all strides in a single trial by the mean stride time of that trial and multiplying by 100%. A stride in the gait cycle is measured from heel strike to heel strike of the same foot. The right and left strides were averaged.

### Neuropsychological Assessment

The Trail Making Test (TMT) and the Centre for Epidemiological Studies – Depression Scale (CES-D) were used to assess cognitive function and mood characteristics commonly associated with cerebrovascular changes and gait. These tests were administered in a quiet room by a trained investigator and took approximately 15 min to complete. The TMT is a measure of higher-level cognitive function such as task switching and sustained attention which are included in the broad term of executive function (Arbuthnott and Frank, 2000). We reported on time to complete the TMT part B. The CES-D scale is a 20-item ordinal scale, scored out of 60, that screens for depressive symptoms. The measure has been shown to be valid and reliable in identifying depressive symptoms in the healthy community-dwelling population (Beekman et al., 1997).

### MRI Acquisition

We acquired neuroimaging using a 3-Tesla MRI system (Achieva, Philips Healthcare, Best NL) with a body coil transmitter and an 8-channel head coil receiver. Participants refrained from vigorous exertion, such as exercise, on the day of the MRI scan. High-resolution three-dimensional T1-weighted images were acquired with repetition time (TR) = 9.5 ms, echo time (TE) = 2.3 ms, flip angle = 8°, voxel dimensions = 0.9 × 0.7 × 1.2 mm^3^, and field of view (FOV) = 240 × 191 × 168 mm^3^. Perfusion imaging was acquired using a pseudo-continuous arterial spin labeling (ASL) sequence, described by the following. A train of radio frequency pulses [duration = 0.5 ms, flip angle = 18°, inter-pulse pause = 0.5 ms] with a balanced gradient scheme was applied over a label duration of 1650 ms. The labeling plane was prescribed perpendicular to the internal carotid artery, at least 5 mm distal to the carotid bifurcation as visualized by a phase-contrast scout image. Thirty control and tag volume pairs were acquired by multi-slice two-dimensional echo planar imaging with TR = 4000 ms, TE = 9.6 ms, flip angle = 90°, in-plane resolution = 3 × 3 mm^3^, FOV = 192 × 192 mm^3^, slice thickness = 5 mm, number of slices = 18 (no gap), and post-label delay = 1600 ms for the first slice and ascending for subsequent slices. A proton density-weighted ASL reference volume was acquired with TR = 10 s, but otherwise identical parameters to the ASL sequence, to estimate the equilibrium magnetization, extract a receiver coil sensitivity profile, and aid in CBF quantification. Two-dimensional T2-weighted images with fluid-attenuated inversion recovery (FLAIR) were acquired with TR = 9000 ms, TE = 125 ms, TI = 2800 ms, flip angle = 90°, voxel dimensions = 0.4 × 0.4 × 3 mm^3^, FOV = 240 × 240 × 156 mm^3^.

### MRI Processing

We used the Oxford Centre for Functional MRI of the Brain Software Library (package fsl-4.1)^1^ to process images. Brain extraction (Smith, 2002) and segmentation (Zhang et al., 2001) tools isolated gray matter, white matter, and cerebrospinal fluid using the T1-weighted image. CBF was calculated from the ASL control-tag difference images after motion correction (Shirzadi et al., 2018) and spatial smoothing with a 5 mm Gaussian kernel. CBF levels were adjusted slice-by-slice to account for the incremental post-label delay and calibrated by a proton density-weighted image (Alsop et al., 2015). Gray matter segmented CBF estimates were calculated for the whole brain and 4 subcortical regions of interest (ROI) as defined by the Montreal Neurological Institute atlas, averaged between the right and left hemispheres: the putamen, pallidum, thalamus, and hippocampus. CBF estimates were also calculated for the frontal cortical region. Whole brain WMH volume was quantified by segmenting hyperintense voxels in the white matter on FLAIR imaging using in-house software. Briefly, the FLAIR images were corrected for signal inhomogeneity, co-registered to T1 for enhanced removal of non-brain tissue, and passed through an anisotropic diffusion filter with minimal smoothing (Gibson et al., 2010). Unambiguous hyperintense voxels were removed to optimize a two-class clustering algorithm on the remaining tissue that distinguished between brain and cerebrospinal fluid on a graded scale. Hyperintense voxels were identified as those deemed to be outliers in both classes (Gibson et al., 2010). The resulting image was then masked against a white matter template (Montreal Neurological Institute) that had been registered to the FLAIR image and thresholded at a white matter probability of 0.5. All lesions identified by the automated process were confirmed visually; manual editing was minimal.

### Statistical Analysis

We performed statistical analyses using R (v.3.3.1) (R Core Team, 2019). Significance was set at *p* < 0.05. First, we examined the effect of potential confounding variables age, sex, and height on CBF and gait. Associations for age with CBF and gait variables were assessed by Pearson correlation. Sex differences in CBF and gait variables were assessed by analysis of covariance (ANCOVA), which adjusted for age in CBF analyses, and age and height in gait analyses. All subsequent analyses for the association of subcortical CBF and gait were adjusted for age, sex, and height. We began by testing the effect for each gait characteristic (independent variable) on CBF in the 4 subcortical ROI (dependent variables) in an omnibus approach using a multivariate analysis of variance (MANOVA) (Fox and Weisberg, 2019). A separate MANOVA model was used for each gait variable. Then, we examined each ROI individually using adjusted multiple regression models. Due to the substantial evidence linking cortical regions, in particular frontal cortical regions, to dual-task gait, we also tested associations between frontal CBF and gait using multiple regression models. Finally, sensitivity analyses explored whether the associations between gait and CBF were influenced by WMH volume, executive function (TMT-B scores), or depression symptoms (CES-D scores) using separate multiple regression models. WMH volume was log-transformed within the regression model. In each sensitivity analysis, the confounding effect of the additional variable was assessed by examining the change in the model’s adjusted r^2^, as well as in the effect size (i.e., the parameter estimate) for the gait variable in the model. A change of 10% was interpreted as a threshold for confounding (Lee, 2014). We calculated Cohen’s *f* to assess the effect size of the strongest relationship between gait and subcortical CBF, and used this effect size to assess the statistical power within this exploratory study.

## Results

Thirty-two older adults (16 females) participated in this study. Demographic, clinical, and neuroimaging characteristics are reported in Table 1. Thalamic CBF was negatively correlated with age (*r* = −0.37, *p* = 0.037), but while CBF in the remaining ROI all had negative correlation coefficients with respect to age (range: -0.17 to -0.31), none of the associations were statistically significant. Further, no gait variables were associated with age, although a trend was observed for stride time CoV, which had a weak direct correlation with age (*r* = 0.31, *p* = 0.08). When adjusting for age and height, gait variables were mostly similar between males and females. The sole exception was that males exhibited longer stride length during the self-paced task (Table 2). In contrast, sex differences in CBF were prevalent. When adjusting for age, females presented with greater CBF than males in the pallidum, thalamus, and frontal ROI (Table 3).

**TABLE 1:** Demographic and clinical characteristics of participants.

**Variable**	
Age, years	72(59−79)
Sex – male, n (%)	16(50)
Education, years	18(10−25)
Height, m	1.68(1.51−1.82)
Body mass index, kg/m^2^	27.7(19.5−40.8)
**Comorbidities**	
Hypertension, n (%)	18(56)
Dyslipidemia, n (%)	14(44)
Type 2 diabetes mellitus, n (%)	4(12)
**Cognition and Mood**	
Trail Making Test B, normative score ^a^	84(44−242)
CES-D, total score (max = 60) ^a^	8.5(2−25)
**Neuroimaging**	
Cerebral blood flow, mL/100 g/min	
Hippocampus	51(28−73)
Pallidum	30(18−53)
Putamen	48(33−81)
Thalamus	47(15−92)
Frontal	53(26−81)
White matter hyperintensity volume, mL	6.4(0.7−43.5)

*Continuous data are presented as median (range) and categorical data are presented as count (percentage). CES-D = Centre for Epidemiologic Studies Depression Scale. N = 32. a, N = 30.*

**TABLE 2:** Sex differences in self-paced and dual-task gait variables adjusted for age and height.

**Variable**	**Males**	**Females**	***p*-value**
Stride velocity, cm/s	127.2(6.3)	115.7(16.2)	0.21
Stride length, cm	142.2(7.3)	122.9(10.2)	0.033
Stride time, s	1.1(0.0)	1.1(0.1)	0.87
Stride time CoV,%	2.2(0.9)	2.4(1.1)	0.99
DT Stride velocity, cm/s	119.5(16.7)	105.1(19.1)	0.29
DT Stride length, cm	138.8(11.2)	118.2(11.2)	0.61
DT Stride time, s	1.2(0.1)	1.1(0.2)	0.11
DT Stride time CoV,%	4.6(2.4)	3.4(2.3)	0.23

*Data are unadjusted mean (sd). P-values represent sex difference adjusted for age and height using ANCOVA. CoV, coefficient of variation; DT, dual-task. N = 16 males and 16 females.*

**TABLE 3:** Sex differences in regional cerebral blood flow adjusted for age.

**Variable**	**Males**	**Females**	***p*-value**
Hippocampus CBF, mL/100 g/min	51.2(12.5)	50.1(12.6)	0.77
Pallidum CBF, mL/100 g/min	28.6(4.7)	36.4(10.7)	0.012
Putamen CBF, mL/100 g/min	48.3(7.1)	55.1(15.5)	0.13
Thalamus CBF, mL/100 g/min	42.3(13.2)	60.8(18.5)	0.002
Frontal CBF, mL/100 g/min	44.9(8.9)	58.1(15.3)	0.006

*Data are unadjusted mean (SD). P-value represents sex difference adjusted for age using ANCOVA. N = 16 males and 16 females.*

**FIGURE 1:**
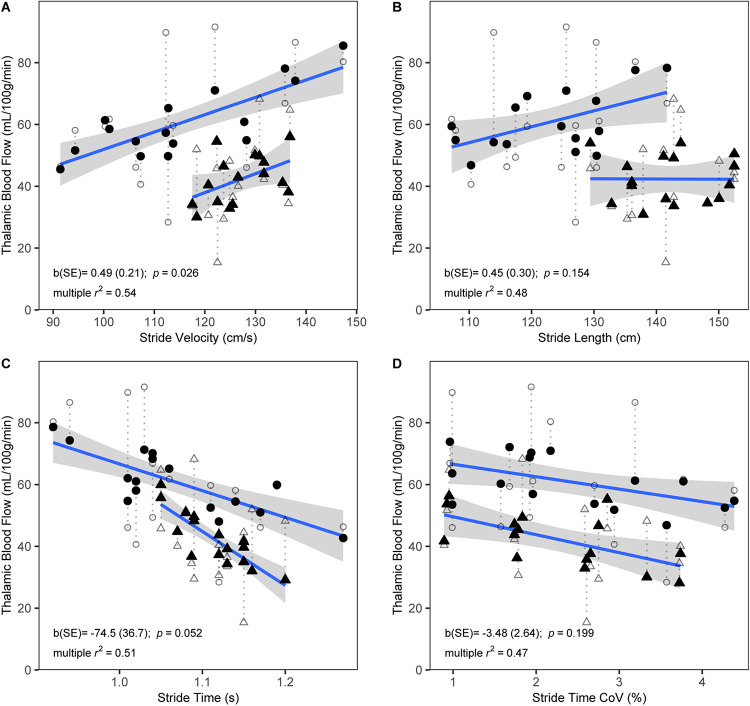
Scatterplots showing bivariate associations of thalamic CBF with self-paced stride velocity **(A)**, stride length **(B)**, stride time **(C)**, and stride time coefficient of variation (CoV) **(D)**. Raw CBF (open symbols) and fitted CBF from multiple regression models that included age, sex, and height as covariates (closed symbols) are shown. Dotted lines link corresponding raw and fitted values. Fitted CBF is shown separately for males (triangles) and females (circles). Regression lines and standard errors are shown for the association of the fitted CBF with stride variables, separately for males and females. Parameter estimates (b) and standard error (SE) from the multiple regression models for the corresponding gait variables are shown. R-squared values represent explained variance of the full multiple regression models. *N* = 32.

During self-paced gait, no overall effect was observed for the association between gait and subcortical CBF (Table 4). In separate regression analyses for each ROI, however, gait velocity was directly related to higher thalamic CBF (*p* = 0.026; Table 5). Trends were also observed denoting inverse associations for gait stride time with thalamic CBF (*p* = 0.052), as well as pallidum CBF (*p* = 0.078) (Table 5). Across the four gait variables, the largest effects were consistently observed in relation to thalamic CBF (Table 5 and Figure 1). In the dual-task condition, no association was observed between the gait variables and subcortical CBF when adjusting for age, sex, and height, either in the omnibus approach (Table 4) or the individual multiple regressions (Table 5). Furthermore, no significant relationship between frontal CBF and gait variables from either self-paced or dual-task challenges was observed (Table 6).

**TABLE 4:** Omnibus association between subcortical CBF and gait stride variables adjusted for age, sex, and height.

	**Velocity (cm/s)**			**Stride Length (cm)**			**Stride Time (s)**			**Stride Time CoV (%)**		
	**F (4,27)**	**Pillai**	***p***	**F (4,27)**	**Pillai**	***p***	**F (4,27)**	**Pillai**	***p***	**F (4,27)**	**Pillai**	***p***
Self-paced	1.36	0.18	0.28	1.29	0.18	0.30	1.32	0.18	0.29	2.08	0.26	0.12
Dual-task	0.63	0.09	0.65	0.80	0.40	0.54	0.27	0.04	0.89	0.42	0.06	0.79

*MANOVA output. Adjusted for age, sex, and height. N = 32.*

**TABLE 5:** Parameter coefficients for the association between subcortical CBF and gait stride variables adjusted for age, sex, and height.

	**Velocity (cm/s)**		**Stride Length (cm)**		**Stride Time (s)**		**Stride Time CoV (%)**	
	**b (SE)**	***t***	**b (SE)**	***t***	**b (SE)**	***t***	**b (SE)**	***t***
**Self-Paced**								
Hippocampus	0.05(0.20)	0.27	−0.05(0.27)	–0.20	−24.6(33.9)	–0.73	0.40(2.37)	0.17
Pallidum	0.16(0.12)	1.28	0.04(0.17)	0.25	−37.7(20.6)	–1.83	0.83(1.51)	0.55
Putamen	0.30(0.18)	1.70	0.30(0.25)	1.19	−42.3(31.0)	–1.36	−1.53(2.20)	–0.69
Thalamus	0.49(0.21)	2.36*	0.45(0.30)	1.47	−74.5(36.7)	–2.03	−3.48(2.64)	–1.32
**Dual-Task**								
Hippocampus	−0.04(0.15)	–0.30	−0.13(0.25)	–0.52	8.0(19.4)	0.42	0.68(1.00)	0.68
Pallidum	−0.12(0.09)	–1.28	−0.22(0.16)	–1.43	12.4(12.2)	1.02	−0.24(0.64)	–0.37
Putamen	−0.08(0.14)	–0.57	−0.15(0.23)	–0.65	12.3(18.0)	0.68	0.31(0.94)	0.33
Thalamus	−0.13(0.17)	–0.75	−0.24(0.29)	–0.85	14.5(22.2)	0.65	0.27(1.16)	0.23

Adjusted for age, sex, and height; b = unstandardized parameter estimate; SE = standard error; CoV = coefficient of variation. N = 32. Value of b reflects the change in CBF, in mL/100g/min, associated with a one-unit change in the gait variable. *p < 0.05.

**TABLE 6:** Parameter coefficients for the association of frontal CBF and gait stride variables adjusted for age, sex, and height.

	**Velocity (cm/s)**		**Stride Length (cm)**		**Stride Time (s)**		**Stride Time CoV (%)**	
	**b (SE)**	***t***	**b (SE)**	***t***	**b (SE)**	***t***	**b (SE)**	***t***
Self-paced	0.23(0.19)	1.18	0.24(0.27)	0.89	−33.4(33.7)	–0.99	−1.36(2.36)	–0.58
Dual-task	−0.06(0.15)	–0.39	−0.22(0.25)	–0.89	4.9(19.5)	0.25	0.26(1.01)	0.26

*Adjusted for age, sex, and height, b = unstandardized parameter estimate; SE = standard error; CoV, coefficient of variation. N = 32. Value of b reflects the change in CBF, in mL/100 g/min, associated with a one-unit change in the gait variable.*

Sensitivity analyses were restricted to the association between thalamic CBF and self-paced gait velocity described above. In the model that included WMH volume, the explained variance of CBF (i.e., adjusted r^2^) was 0.45 and the effect size [i.e., parameter estimate (SE)] for the association between stride velocity and thalamic CBF was 0.47 (0.21) mL/100 g/min per cm/s (*p* = 0.035). This equates to a 3% reduction in explained variance and a 4% reduction in the effect size relative to the model without WMH burden. In the model that included the scaled TMT-B score, as a marker of executive function, the adjusted *r*^2^ was 0.49 and the gait-CBF effect size was 0.52 (0.23) mL/100 g/min per cm/s (*p* = 0.031). This equates to a 5% increase in the explained variance and a 6% increase in effect size relative to the model without TMT-B. Finally, in the model that included the CES-D score, as an index of depression symptoms, the adjusted *r*^2^ was 0.49 and the stride velocity-CBF effect size was 0.55 (0.22) mL/100 g/min per cm/s (*p* = 0.021); equivalent to a 5% increase in the explained variance and 12% increase in the effect size.

Cohen’s *f* for the relationship between stride velocity and thalamic CBF was 0.21, suggesting a moderate standardized local effect size. A *post hoc* calculation, given our sample size, determined the study’s power to detect this size of effect to be 0.44.

## Discussion

This study investigated associations between subcortical CBF and gait in older adults. While there was no overarching association for CBF across the four chosen subcortical ROI with gait, two key observations were brought to light. First, an effect within the individual thalamic ROI was noted, in which lower CBF was associated with lower stride velocity during gait at a self-selected pace. Second, a sensitivity analysis found that accounting for depressive symptoms strengthened the association between thalamic CBF and gait velocity. Surprisingly, no association was observed between subcortical or frontal CBF and dual-task gait characteristics. This is the first study to identify an association between thalamic CBF and gait, providing further support to the notion that subcortical vascular health is an important biomarker of functional decline in older adults.

The thalamus is a central action hub in the brain with input and output tracts between multiple brain regions. A group of nuclei called the motor thalamus is positioned between the motor areas of the cortex and subcortical structures, such as the pallidum and putamen, and processes inputs from the two networks (Roland, 1984). The functional integration of thalamic circuits into gait performance has been identified previously. Su and colleagues observed that thalamus atrophy mediates the association between SVD burden and poorer performance on 3-m walk and chair stand tests (Su et al., 2018). Further, the presence of thalamic infarcts, in particular, and WMH in the sublobar region have been independently linked to slower gait velocity (de Laat et al., 2010). Our work aligns with these findings suggesting that physiological changes occurring at the thalamus may alter the brain network responsible for gait. Cerebrovascular changes in the brain have previously been implicated in lower extremity functional deficits. Lower resting MCA blood velocity is associated with slower gait speed and poorer lower extremity function (Ezzati et al., 2017). In addition, reduced neurovascular coupling in the MCA perfusion territory is associated with slow gait speed and falls amongst older adults (Sorond et al., 2011; Jor’dan et al., 2017). Notably, these studies are grounded in the paradigm that MCA dysfunction reflects impaired perfusion in the motor and frontal cortices, while the thalamus is primarily perfused by branches of the posterior cerebral arteries. Future work involving the differential effects between anterior and posterior cerebral circulations may help elucidate mechanisms linking SVD to motor impairment.

Reduced CBF with aging has been largely viewed as an indicator of decreased metabolism (DeCarli et al., 1995; Aanerud et al., 2012) and/or regional atrophy (Appelman et al., 2008; Alosco et al., 2013). Importantly, however, Chen and colleagues observed a clear distinction between brain regions showing the greatest hypoperfusion and regions showing the greatest atrophy (Chen et al., 2011). This divergence was particularly apparent when looking at subcortical CBF and tissue volume (Chen et al., 2011). These findings suggest altered hemodynamics in older adults may contribute to functional impairment independent of structural changes in the brain. Our analyses were focused on the subcortical regions that contribute to frontal-subcortical connections. The hippocampus was also defined *a priori* because it influences frontal-subcortical functions in relation to spatial navigation and has shown to be associated with gait (Lithfous et al., 2013; Beauchet et al., 2015), although we did not observe any association between gait measures and hippocampal CBF. In the longitudinal Health Aging and Body Composition study, however, hippocampal atrophy was associated with both gait slowing and onset of cognitive impairment over 14 years (Rosso et al., 2017). These discrepancies in the association between gait measures and hippocampal integrity at a single time point speak to the importance of characterizing gait changes over time (Rosano et al., 2008; Ezzati et al., 2015). We posited that the underlying cognitive component linking CBF and gait would have been most sensitive to dual-task effects (Gatouillat et al., 2015), yet no association between CBF and dual-task cost was observed. Given the likely involvement of cortical tissue, most notably frontal cortical regions associated with dual-task effects on gait, we felt it pertinent to add a secondary analysis of frontal CBF and gait. Our results did not demonstrate any significant associations between frontal CBF and self-paced or dual-task gait. The lack of findings may be the result of having an under-powered sample, as parameter estimates were pointing in the hypothesized direction (i.e., faster gait and lower variability related to higher CBF). It is also possible that our highly educated sample did not perceive the dual-task (i.e., serial 7’s from 100) to be sufficiently challenging. Under dual-task walking conditions individuals with MCI have shown significant dual-task deficits while cognitively healthy controls tend to perform within clinically normal levels (Montero-Odasso et al., 2012). Another potential explanation is that resting CBF in frontal regions may not be an appropriate indicator of dual-task walking. Jor’dan et al. (2017) found that increases in CBF during an executive task were associated with increased neural activity in the dorsal attentional and executive networks, and that these measures were significantly associated with gait speed. The association between task-related neuronal activation, change in CBF, and walking speed shows that CBF works to meet the metabolic demands of the regions responsible for gait, particularly the executive networks. Gatouillat et al. (2015) revealed that CBF in the MCA significantly increases to meet the demands of dual-task walking, and that these changes in CBF were associated with gait changes between single and dual-task. These data suggest that task CBF may be a better marker of dual-task gait performance than resting CBF.

Pugh and Lipsitz suggested that key features of the geriatric syndrome, including cognitive decline, mood disorders and gait impairment, are related to damage in the frontal-subcortical circuits caused by SVD (Pugh and Lipsitz, 2002). Sensitivity analyses revealed that the association between thalamic CBF and stride velocity was accentuated when accounting for depressive symptoms (indexed by CES-D score). While vascular depression has been linked to slow gait in older women (Paulson and Lichtenberg, 2013), our results suggest that the relationship between thalamic CBF and stride velocity was not related to pathological processes associated with mood disorders. Indeed, if a relationship between mood, gait, and thalamic CBF existed, the effect size between gait and CBF would have been attenuated when adding CES-D to the model. Notably, our study did not include participants with any major depressive disorder. In addition, they were well-educated and had relatively low WMH burden as a group, thus our findings may be more applicable to a healthy population and not reflective of the co-incident expression of geriatric syndrome.

Cognitive and gait impairments often coexist, and gait slowing and increased variability have been shown to indicate underlying brain changes (particularly WMH volume) and predict long term cognitive decline (Rosano et al., 2007; Srikanth et al., 2009; Mielke et al., 2013; Rosso et al., 2014; MacDonald et al., 2017; Montero-Odasso et al., 2018). That the association between subcortical CBF and self-paced stride velocity was still present when controlling for TMT (part B) scores and WMH suggests that at least a portion of the association is not mediated by higher order processing or structural damage. TMT has previously been shown to be sufficiently sensitive to characterize the association between executive dysfunction and gait (Coppin et al., 2006), however, associations between CBF and gait that are mediated by executive function may be limited to cortical regions, such as the prefrontal cortex (Manor et al., 2016). Due to our small sample size we only conducted our sensitivity analyses with models showing significant independent relationships. It is possible that these features of aging have independent pathological processes and that the mechanisms underlying the associations between resting CBF in subcortical ROI and self-selected walking pace may be separate from those linking cognitive decline and WMH to gait.

This study benefited from the acquisition of robust, objective measures of CBF and gait. Our small sample size may have been a limiting factor for uncovering significant associations between individual ROI and gait, however, the observed relationships were trending in the expected direction. Furthermore, our sample did not have moderate to severe SVD or cognitive impairments, which may have resulted in ceiling effects when assessing gait variability, and dual-task gait. Variability remains low under self-paced walking conditions and it is possible that our dual-task paradigm was not taxing enough to elicit gait changes in our sample. Assuming a similar true effect size to that found between thalamic CBF and gait velocity for the other subcortical ROI, or even for the frontal ROI (i.e., Cohen’s *f* = 0.21), the small sample resulted in an underpowered study (Power = 0.44). Consequently, there remains a high probability of type II error that we were able to detect associations that are posited to exist within the frontal-subcortical connection hypothesis of functional decline. Another issue involves the number of statistical comparisons conducted within our main analysis. As previously mentioned, our sample size was underpowered to achieve conservative significance thresholds for the high number of variables and covariates and we propose that this novel study be interpreted in an exploratory manner to help guide future work in this area. Finally, the study was not designed to determine causation between CBF and gait, but both are recognized as indicators of potential future decline (e.g., dementia, falls) (Wierenga et al., 2014; Montero-Odasso et al., 2017).

## Conclusion

In this study, we identified a direct relationship between gait and subcortical blood flow within the thalamus. This preliminary association between thalamic CBF and gait provides further support to the notion that subcortical vascular health alone, as well as within the context of frontal-subcortical disconnection theory, is an important biomarker of functional decline in older adults. Gait is easily measured, clinically interpretable, and may provide an early window into deleterious changes in the subcortical cerebral vasculature. Strategies aimed at improving blood flow regulation may simultaneously be useful for improving gait and reducing the incidence of falls and fall-related morbidity and mortality in older adults.

## Data Availability Statement

The raw data supporting the conclusions of this article will be made available by the authors, without undue reservation.

## Ethics Statement

This study was reviewed and approved by the Sunnybrook Research Ethics Board. The participants provided their written informed consent to participate in this study.

## Author Contributions

NK contributed to data acquisition, analysis, interpretation, and manuscript preparation and revision. SA contributed to data acquisition, analysis, interpretation, and manuscript revision. EC contributed to data interpretation and manuscript preparation and revision. NA and CG contributed to data interpretation and manuscript revision. BM contributed to conception and design of the work, data analysis, interpretation, and manuscript revision. AR contributed to conception and design of the work, data acquisition, analysis, interpretation, and manuscript preparation and revision. All authors gave final approval of the manuscript to be published, were accountable for all aspects of the work.

## Conflict of Interest

The authors declare that the research was conducted in the absence of any commercial or financial relationships that could be construed as a potential conflict of interest.
